# Prevalence and Risk Factors of Gastrointestinal Disorders in Patients with Rheumatoid Arthritis: Results from a Population-Based Survey in Olmsted County, Minnesota

**DOI:** 10.1155/2011/745829

**Published:** 2011-11-17

**Authors:** Elena Myasoedova, Nicholas J. Talley, Nisha J. Manek, Cynthia S. Crowson

**Affiliations:** ^1^Department of Health Sciences Research, Mayo Clinic College of Medicine, Rochester, MN 55905, USA; ^2^Department of Internal Medicine, Division of Rheumatology, Ivanovo State Medical Academy, Ivanovo, Russia; ^3^Faculty of Health, University of Newcastle, Callaghan, NSW 2308, Australia; ^4^Division of Rheumatology, Mayo Clinic College of Medicine, Rochester, MN 55905, USA

## Abstract

*Objectives*. To compare the prevalence of gastrointestinal (GI) disorders in rheumatoid arthritis (RA) versus non-RA subjects and to describe determinants of GI disorders in RA. *Methods*. The bowel disease questionnaire was completed by RA and non-RA subjects. RA patients also completed the health assessment questionnaire (HAQ). *Results*. The study responders included 284 RA and 233 non-RA subjects. Abdominal pain/discomfort, postprandial fullness, nausea, and stool leakage were significantly more common in RA versus non-RA (odds ratios [OR] = 1.8; 1.9; 4.0; 8.2, resp.). The use of laxatives, proton pump inhibitors, NSAIDs, acetaminophen, and narcotics was more commonly reported in RA versus non-RA (OR = 2.0; 1.7; 3.0; 2.0; 1.9, resp.). Age < 60 and HAQ ≥ 1 were associated with dyspepsia, irritable bowel syndrome, gastroesophageal reflux disease, and GI symptom complex overlap in RA. *Conclusion*. Several upper and lower GI disorders were significantly more prevalent in RA versus non-RA subjects. Age <60 and physical function impairment (HAQ ≥ 1) were associated with GI disorders in RA.

## 1. Introduction

Rheumatoid arthritis (RA) is a chronic autoimmune inflammatory disease affecting ~1% of the adult population and associated with progressive deterioration of joint function, increased morbidity, and mortality [1–3]. Upper gastrointestinal (GI) disease is recognized as one of the major comorbidities in RA leading to a significant increase in the risk of mortality [3–8]. The evidence of increased GI risk in RA is mostly based on the studies of organic GI disorders and GI complications [3–5, 9–12], while functional GI symptoms in RA are much less well characterized [6, 13]. The reasons for the excess GI risk, particularly the risk of functional GI disorders, are not completely understood, and the impact of RA on increased GI morbidity is uncertain. To study the burden of GI disorders in RA patients (particularly functional GI symptoms that may not come to medical attention), we conducted a cross-sectional population-based survey examining the prevalence of functional GI symptoms and syndromes in RA patients versus non-RA subjects. We also aimed to define the risk factors associated with GI disorders in RA.

## 2. Methods 

### 2.1. Study Sampling and Design

Using the resources of the Rochester Epidemiology Project [14], a population-based medical record linkage system that allows ready access to the complete medical records from all community medical providers, we have previously assembled a cohort of residents of Olmsted County, Minnesota, aged ≥ 18 years who first fulfilled ≥4 1987 American College of Rheumatology criteria for RA [15] between 1/1/1980 and 1/1/2008. From this cohort, we identified 493 eligible RA subjects, namely those alive and residing in Olmsted County, Minnesota as of 1/1/2008. Data were collected on rheumatoid factor (RF) tests performed clinically for these patients. 

A similar number (*n* = 493) of non-RA subjects selected from the same population were matched for age, sex, and observational period to the RA patients. Every person in the community ≥18 years of age who was qualified during the defined period regardless of race, ethnicity, or socioeconomic status was equally eligible to participate. The study protocol was approved by Review Boards from Mayo Clinic and Olmsted Medical Center. 

We conducted a population-based cross-sectional survey of the occurrence of GI symptoms among RA and non-RA subjects using the bowel disease questionnaire (BDQ) [16]. RA patients also completed the health assessment questionnaire (HAQ) [17, 18]. The surveys were mailed and returned between August 1, 2008 and December 31, 2008 in Olmsted County, Minn, USA. Subjects who returned a completed questionnaire were considered responders to this survey, and those from whom we did not receive a completed questionnaire were considered nonresponders to the survey.

### 2.2. Bowel Disease Questionnaire

The original BDQ was designed and validated at the Mayo Clinic as a self-report instrument to measure GI symptoms experienced over the prior year and to collect medical history data [16, 19]. The BDQ has been shown to discriminate functional GI disorders and health in the general population [16]. In the present study, we used the shortened version of the questionnaire with 15 questions covering a number of upper and lower GI symptoms, including abdominal pain/discomfort, dysphagia, heartburn, nausea, vomiting, early satiety, postprandial fullness, abdominal bloating, bowel habits, and appearance and frequency of stools. The questionnaire also included questions on current and past drug use (primarily, GI-related medications, analgetics/antipyretics, and nonsteroidal anti-inflammatory drugs (NSAIDs)).

### 2.3. Definitions of GI Syndromes

Based on the responses to the BDQ, the study subjects were classified as having dyspepsia, functional constipation, irritable bowel syndrome (IBS), and/or gastroesophageal reflux disease (GERD) using modified Rome II criteria [20]. *Dyspepsia* was defined based on the presence of 1 or more of the following symptoms: (a) frequent upper abdominal pain occurring ≥1 day a week in the past year; (b) early satiety ≥1 day a week; (c) postprandial fullness ≥1 day a week. We additionally analyzed the number of patients having either dyspepsia or treatment with proton-pump inhibitors, H_2_ antagonists, or gastroprotective agents.* IBS* was defined as abdominal pain or discomfort ≥1 day a month in the past year, and given this, 2 out of the following 3 features: (a) relieved with defecation; (b) onset associated with a change in frequency of stool; (c) onset associated with a change in form (appearance) of stool. *Functional constipation* was defined when the patient had insufficient criteria for IBS but fulfilled 2 or more of the following: (a) <3 bowel movements per week; (b) strain often to have a bowel movement; (c) stools often hard; (d) incomplete evacuation. In addition, we analyzed the number of patients having either functional constipation or using laxatives.* GERD* was defined as weekly or more frequent heartburn or acid regurgitation. If a subject met the criteria for 2 or more of the above disorders, we defined him as having “*GI symptom complex overlap.*” If a subject did not meet the criteria of any of the above disorders, we defined him as having “*no functional GI disorders.*”

### 2.4. Health Assessment Questionnaire (HAQ)

HAQ is a self-report questionnaire which is widely used for assessment of functional disability in patients with rheumatic diseases, particularly, in RA [17, 18]. The HAQ includes questions regarding patient's ability to perform activities of daily living (summarized as HAQ disability index; scored 0–3), and visual analog scales (0–100 mm) for pain and general well-being. As the scores increase, so do the levels of disability, pain, and the level of the overall wellness affected by arthritis, respectively.

### 2.5. Statistical Methods

Descriptive statistics were used to summarize demographics and GI events in both cohorts, as well as RA characteristics in the RA cohort. Comparisons between the two cohorts were performed using Chi-square tests and *t*-tests, as were comparisons between responders and nonresponders. Logistic regression models were used to compare the prevalence of GI symptoms and syndromes between the RA and non-RA cohorts adjusting for age, sex, and obesity (body mass index [BMI] ≥ 30 kg/m^2^) with additional adjustment for the use of NSAIDs. Multivariable logistic regression models were performed considering demographics (age categorized as <60 and ≥60 years; sex), lifestyle factors (smoking, alcohol consumption), obesity, RA characteristics (RF positivity and HAQ score categorized as <1 and ≥1), and NSAIDs use as potential factors associated with GI syndromes in RA.

## 3. Results 

### 3.1. Patients' Characteristics

The study population included 284 RA and 233 non-RA subjects who responded to the survey. This yielded a response rate of 58% and 47% in RA and non-RA cohorts, respectively. The characteristics of both cohorts are summarized in Table 1. The mean (SD) age in the RA cohort was 62.4 (13.4) years (72% female) and in the non-RA cohort was 63.6 (13.4) years (71% female). The mean (SD) BMI estimates were similar in RA patients and non-RA subjects: 29.0 (5.8) and 29.5 (6.8), respectively. There were no significant differences in alcohol and tobacco use between RA and non-RA subjects, but RA subjects were somewhat more likely to have a history of smoking (46% versus 39%, *P* = 0.12; Table 1). 

The mean (SD) RA duration was 10.3 (7.2) years, and 188 (67%) RA patients were positive for RF (Table 2). The mean (SD) HAQ score was 0.6 (0.6). The mean (SD) patient reported pain in the past week due to illness on the visual analogue scale was 28.8 (24.8), and overall wellness affected by arthritis was 23.8 (23.6). Demographics and RA characteristics were similar in RA patients who responded to the survey versus nonresponders to the survey (Table 2).

### 3.2. GI Symptoms and Syndromes in RA versus Non-RA Cohort

GI symptoms were found to be very common in both RA patients and in non-RA subjects. The most common GI complaints in both RA patients and non-RA subjects were abdominal pain/discomfort (18% and 10%, resp.) and postprandial fullness (18% and 10%, resp.; Table 3). However, the prevalence of several GI symptoms was higher in RA patients than in non-RA subjects, even after adjustment for age, sex, and obesity (BMI ≥ 30 kg/m^2^). In particular, RA patients were more likely than non-RA subjects to experience abdominal pain/discomfort (odds ratio [OR] 1.8, 95% confidence interval [CI] 1.1, 3.1), postprandial fullness (OR 1.9, 95% CI 1.1, 3.3), nausea (OR 4.0, 95% CI 1.1, 14.2), and stool leakage (OR 8.2, 95% CI 1.03, 66). The likelihood of dyspepsia in RA versus non-RA subjects approached statistical significance (OR 1.6, 95% CI 0.9, 2.8; *P* = 0.10). No significant differences were found between RA and non-RA subjects for GI syndromes, including functional constipation, IBS, and GERD. The likelihood of having GI symptom complex overlap, as well as the likelihood of having no functional GI disorders, was similar in the RA and non-RA cohorts. The odds ratios for these associations changed only minimally after the additional adjustment for NSAIDs (data not shown).

### 3.3. Medication Usage in RA versus Non-RA Cohort

RA patients were more likely than non-RA subjects to take medications for their GI disorders (Table 3). These included laxatives (OR 2.0, 95% CI 1.1, 3.5) and proton pump inhibitors (OR 1.7, 95% CI 1.1, 2.5). The likelihood of using H_2_ antagonists in RA versus non-RA subjects approached statistical significance (OR 1.6, 95% CI 0.95, 2.7; *P* = 0.08). The proportion of those having dyspepsia or using medications for dyspepsia (namely, proton-pump inhibitors, H_2_ antagonists, or gastroprotective agents) was 1.9-fold higher in RA versus non-RA cohort (Table 3). Not surprisingly, RA patients took more NSAIDs (OR 3.0, 95% CI 1.8, 5.0), acetaminophen (OR 2.0, 95% CI 1.2, 3.4), and narcotic medications (OR 1.9, 95% CI 1.2, 3.0) as compared to non-RA subjects. The intake of other medications (i.e., antispasmodics, antacids, gastroprotective agents, antidiarrheal medications, antidepressants, and calcium channel blockers) was similar in both the RA and non-RA cohorts. After adjusting for age, sex, and obesity, further adjusting for NSAIDs did not substantially change the results (data not shown).

### 3.4. Factors Associated with GI Syndromes in RA

For RA patients, we further examined the associations of demographics (age categorized as <60 and ≥60 years; sex), lifestyle factors (smoking, alcohol consumption), obesity, RA characteristics (RF positivity and HAQ score categorized as <1 and ≥1), and NSAIDs with each of the GI syndromes (dyspepsia, functional constipation, IBS, GERD, and GI symptom complex overlap) using multivariable models assessing all factors of interest simultaneously. The results of these analyses are summarized in Table 4. Dyspepsia, IBS, GERD, and GI symptom complex overlap were more likely to occur in RA patients aged <60 years, than in those who were ≥60 years as well as among the patients with HAQ score ≥1 compared to those with HAQ score <1. RA patients <60 years were less likely to be free of GI disorders than those aged ≥ 60 years. The association of other potential GI risk factors (including gender, obesity, smoking, alcohol consumption, and RF positivity) and NSAIDs use with GI syndromes did not reach statistical significance (*P* > 0.25 for all). 

## 4. Discussion

We report the results of a population-based survey of GI symptoms and syndromes in RA patients compared to matched non-RA subjects from the same population. This study demonstrated the increased prevalence of several upper and lower GI symptoms such as abdominal pain/discomfort, postprandial fullness, nausea, and stool leakage in RA patients as compared to the non-RA subjects. Further, we observed a higher prevalence of GI symptoms occurring at least once a week (i.e., very often) in RA patients compared to non-RA subjects. We have also found the more frequent use of some GI-related medications, particularly laxatives and proton pump inhibitors in RA compared to the non-RA subjects. Our results are concordant with previous observations showing higher prevalence of the dysmotility-like symptoms in some rheumatological disorders including RA [6, 13]. The nature of these functional GI disorders remains uncertain.

One potential reason for this increased burden of functional GI disorders in RA is the effect of antirheumatic medications [9–11, 21–30]. In fact, GI disease in RA has been extensively studied in the context of drug-related upper GI complications, and the adverse effects of antirheumatic medications (particularly, NSAIDs) have been clearly demonstrated [21–30]. However, recent evidence suggests that the implementation of the guidelines for prevention of NSAID-gastropathy may lead to the decrease in NSAID-associated GI events [12, 31, 32]. Concordantly, we did not find the association of NSAIDs use with GI syndromes in our study. 

Alternatively, GI disorders may be a part of the clinical spectrum of RA disease [5, 33]. In this study, we observed an association of physical function impairment (i.e., higher HAQ score) with a number of GI syndromes. While disability itself can cause difficulties with usual activities in RA patients, this association raises the possibility that active systemic inflammation may play a role in the development of GI disorders. Others have observed that RF seropositive RA patients are more likely to develop GI bleeding compared to RF seronegative patients [11]. Although our study did not demonstrate a significant association between RF positivity and GI syndromes, this may be due to the lack of power and requires further elucidation.

Our observation of higher likelihood of GI syndromes in RA patients aged <60 years than in those ≥60 years indirectly corroborates with the previous findings on the lower frequency of GI symptoms in older ages in general population and may be linked to changes in visceral sensitivity with aging [34, 35]. The alternative explanation could be a possibility of confounding by indication when patients ≥60 years at risk for GI complications receive more gastroprotective treatment, thus leading to the lower frequency of GI disorders. Consistent with previous studies of the general population [36, 37], smoking and alcohol consumption were not significantly associated with GI disorders. 

The results of this study should be interpreted in the context of several limitations. First, despite the large number of subjects in both the RA and non-RA cohorts, statistical power is limited for some comparisons due to the infrequency of some GI events. Thus, we may not have been able to demonstrate statistical significance for some important clinical differences. Secondly, due to the cross-sectional design, we captured only prevalent GI symptoms. Thus, some GI symptoms with short duration could have been missed. However, considering that GI disorders are predominantly chronic conditions and that subjects were asked to report GI symptoms during the past year, we believe that the cross-sectional design was appropriate and provided reliable information on GI symptoms in our study. Third, the results of the study are based on patient self-report, which is subject to recall bias. However, this bias is likely to be nondifferential as the limitation applied equally to both the RA and non-RA cohorts. Further, the use of a standardized validated questionnaire which previously demonstrated adequate performance in the general population is likely to minimize this weakness. Fourth, there is a possibility of nonresponse bias, but no significant differences in the characteristics of survey respondents versus nonrespondents to the survey were found. Fifth, during this survey, we did not collect information on serious GI complications (including GI ulcer, GI bleed, GI perforation, and bowel obstruction), antirheumatic medications (including biologics), antiosteoporotic medications, and comorbidities for each patient. However, from our other studies of this cohort, we know that only a small percentage (roughly 16%) have used biologics, so it is unlikely that the use of biologics has a significant impact on our results. Epidemiology and impact of serious GI events in RA is subject of our ongoing study. Lastly, the population of Olmsted County, Minnesota is predominantly Caucasian suggesting that the results of the study may not be generalizable to more ethnically diverse populations. Notable strengths of the study include a population-based study design with a comparison cohort of non-RA subjects from the same population and the use of a validated questionnaire. 

In conclusion, several upper and lower GI disorders are significantly more prevalent in RA patients compared to the general population. RA patients are more likely to take analgesic medications, laxatives, and proton-pump inhibitors than non-RA subjects. Younger age (<60 years) and physical function impairment (HAQ score ≥1) are associated with some GI syndromes in RA. This raises the possibility that the impact of RA on GI disease is more widespread than previously believed. Together these findings suggest that there is need for the increased awareness of the GI symptoms in RA. More research is needed to further examine the nature of GI disorders in RA.

## Figures and Tables

**Table 1:** Characteristics of RA and non-RA cohorts.

Variable	RA (*n* = 284)	Non-RA (*n* = 233)	*P* value*
Mean age, years (SD)	62.4 (13.4)	63.6 (13.4)	0.39
Female, *n* (%)	204 (72)	165 (71)	0.80
Mean BMI, kg/m^2^ (SD)	29.0 (5.8)	29.5 (6.8)	0.69
Alcoholic drinks, number per week			0.80
<1/week, *n* (%)	192 (68)	151 (65)	
1–6/week, *n* (%)	70 (25)	62 (27)	
≥7/week, *n* (%)	22 (8)	20 (9)	
Ever Smoker, *n* (%)	129 (46)	92 (39)	0.12

RA: rheumatoid arthritis; SD: standard deviation; BMI: body mass index.

*P value indicates the differences between RA and non-RA cohorts.

**Table 2:** Demographics and RA characteristics in survey responders and nonresponders to the survey among patients with rheumatoid arthritis.

Variable	Responders to the survey (*N* = 284)	Nonresponders to the survey (*N* = 209)	*P* value*
Mean age on the date of response, years (SD)	62.4 (13.4)	61.0 (15.4)	0.16
Female, *n* (%)	204 (72)	155 (74)	0.57
Mean duration of RA, years (SD)	10.3 (7.2)	9.8 (6.9)	0.55
RF positive, *n* (%)	188 (67)	141 (68)	0.79

*P value indicates the differences between survey responders and nonresponders to the survey.

Abbreviations: RA: rheumatoid arthritis; SD: standard deviation; RF: rheumatoid factor.

**Table 3:** Gastrointestinal complaints and medications in RA and non-RA cohorts^a^.

Variable	RA (*n* = 284)	Non-RA (*n* = 233)	Odds ratio^b^ (95% CI) adjusting for age, sex, and obesity (BMI ≥ 30 kg/m^2^)
*GI symptoms (≥1 day per week)*			
Abdominal pain/discomfort	52 (18)	24 (10)	**1.8 (1.1, 3.1)**
Early satiety^c^	30 (12)	19 (9)	1.3 (0.7, 2.3)
Postprandial fullness^d^	47 (18)	22 (10)	**1.9 (1.1, 3.3)**
Nausea	15 (6)	3 (1)	**4.0 (1.1, 14.2)**
Stool leakage	10 (4)	1 (0.4)	**8.2 (1.03, 66)**

*GI syndromes*			
Dyspepsia	43 (16)	22 (10)	1.6 (0.9, 2.8)
- Dyspepsia or treatment with proton-pump inhibitors, H_2_ antagonists, or gastroprotective agents	132(46)	72 (31)	**1.9 (1.3, 2.7)**
Functional constipation	82 (29)	67 (29)	1.1 (0.7, 1.6)
- Functional constipation or laxative use	116 (41)	84 (36)	1.3 (0.9, 1.9)
Irritable bowel syndrome	82 (29)	59 (26)	1.1 (0.7, 1.6)
Gastroesophageal reflux disease	41 (14)	26 (11)	1.4 (0.8, 2.3)
GI symptom complex overlap	50 (18)	28 (12)	1.4 (0.8, 2.4)
No functional GI disorders	103 (36)	94 (40)	0.9 (0.6, 1.2)

*Medications*			
Laxatives	43 (16)	20 (9)	**2.0 (1.1, 3.5)**
Proton-pump inhibitors	89 (32)	48 (21)	**1.7 (1.1, 2.5)**
Gastroprotective agents (sucralfate, misoprostol)	9 (3.2)	2 (0.9)	3.7 (0.8, 17.6)
H_2_ antagonists	46 (16)	25 (11)	1.6 (0.95, 2.7)
NSAIDs, ≥7 tab./cap per week	73 (26)	26 (11)	**3.0 (1.8, 5.0)**
Acetaminophen, ≥7 tab. per week	58 (21)	26 (11)	**2.0 (1.2, 3.4)**
Narcotic pain medications	68 (24)	34 (15)	**1.9 (1.2, 3.0)**
Iron supplements	49 (18)	27 (12)	1.6 (0.9, 2.6)

^a^All data are shown as *n* (%).

^b^Odds ratio compares RA to non-RA; significant (*P* < 0.05) odds ratios are shown in bold.

^c^Defined as inability to finish a regular-sized meal.

^d^Defined as feeling too full after a regular-sized meal.

Abbreviations: GI: gastrointestinal; RA: rheumatoid arthritis; NSAIDs: nonsteroid anti-inflammatory drugs.

**Table 4:** Multivariable models of variables^#^ associated with GI syndromes in RA patients.

Variables	Dyspepsia	Functional constipation	IBS	GERD	GI symptom complex overlap	No functional GI disorders
Age < 60 yrs	**2.5 (1.2, 5.4)**	0.6 (0.4, 1.1)	**1.9 (1.1, 3.4)**	**2.8 (1.3, 6.0)**	**2.2 (1.1, 4.4)**	**0.5 (0.3, 0.9)**
Female sex	2.3 (0.8, 6.6)	0.6 (0.3, 1.1)	1.4 (0.7, 2.8)	0.6 (0.3,1.4)	1.4 (0.6, 3.3)	1.3 (0.7, 2.4)
HAQ ≥1	**2.4 (1.1, 5.1)**	0.6 (0.3, 1.1)	**2.4 (1.3, 4.5)**	**3.2 (1.5, 7.1)**	**3.1 (1.5, 6.3)**	0.7 (0.4, 1.3)
RF positivity	1.0 (0.4, 2.1)	1.0 (0.6, 1.9)	1.2 (0.7, 2.2)	1.2 (0.5, 2.6)	1.0 (0.5, 2.1)	0.8 (0.5, 1.3)
Obesity (BMI ≥ 30 kg/m^2^)	1.1 (0.5, 2.4)	1.0 (0.6, 1.9)	0.9 (0.5, 1.6)	0.9 (0.4, 1.9)	0.9 (0.4, 1.8)	1.1 (0.6, 1.8)
NSAIDs use	1.4 (0.7, 3.2)	0.8 (0.4, 1.5)	1.5 (0.8, 2.8)	0.8 (0.4, 1.9)	1.2 (0.6, 2.5)	0.9 (0.5, 1.7)
Smoking	1.3 (0.6, 2.8)	1.1 (0.6, 2.0)	1.0 (0.6, 1.8)	0.5 (0.2, 1.1)	0.8 (0.4, 1.6)	1.0 (0.6, 1.7)
Alcohol use	2.0 (0.6, 6.8)	1.0 (0.4, 2.6)	0.4 (0.1, 1.5)	1.3 (0.3, 5.0)	1.6 (0.5, 5.2)	1.7 (0.7, 4.3)

^#^Significant (*P* < 0.05) odds ratios are shown in bold.

Abbreviations: GI: gastrointestinal; RA: rheumatoid arthritis; IBS: irritable bowel syndrome; GERD: gastroesophageal reflux disease; HAQ: health assessment questionnaire; RF: rheumatoid factor; BMI: body mass index; NSAIDs: nonsteroidal anti-inflammatory drugs.
